# Gut eukaryotic communities in pigs: diversity, composition and host genetics contribution

**DOI:** 10.1186/s42523-020-00038-4

**Published:** 2020-05-07

**Authors:** Yuliaxis Ramayo-Caldas, Francesc Prenafeta-Boldú, Laura M. Zingaretti, Olga Gonzalez-Rodriguez, Antoni Dalmau, Raquel Quintanilla, Maria Ballester

**Affiliations:** 1 Animal Breeding and Genetics Program, Institute of Agrifood Research and Technology (IRTA), Torre Marimon, 08140 Caldes de Montbui, Spain; 2 GIRO, IRTA, Torre Marimon, 08140 Caldes de Montbui, Spain; 3 Centre for Research in Agricultural Genomics, CSIC-IRTA-UAB-UB Consortium, Bellaterra, Spain; 4 Animal Welfare Subprogram, IRTA, 17121 Monells, Spain

**Keywords:** Gut eukaryotes, Fungi, Protists, Pig, Host genetics

## Abstract

**Background:**

The pig gut microbiome harbors thousands of species of archaea, bacteria, viruses and eukaryotes such as protists and fungi. However, since the majority of published studies have been focused on prokaryotes, little is known about the diversity, host-genetic control, and contributions to host performance of the gut eukaryotic counterparts. Here we report the first study that aims at characterizing the diversity and composition of gut commensal eukaryotes in pigs, exploring their putative control by host genetics, and analyzing their association with piglets body weight.

**Results:**

Fungi and protists from the faeces of 514 healthy Duroc pigs of two sexes and two different ages were characterized by 18S and ITS ribosomal RNA gene sequencing. The pig gut mycobiota was dominated by yeasts, with a high prevalence and abundance of *Kazachstania* spp. Regarding protists, representatives of four genera (*Blastocystis, Neobalantidium, Tetratrichomonas and Trichomitus*) were predominant in more than the 80% of the pigs. Heritabilities for the diversity and abundance of gut eukaryotic communities were estimated with the subset of 60d aged piglets (*N* = 390). The heritabilities of α-diversity and of the abundance of fungal and protists genera were low, ranging from 0.15 to 0.28. A genome wide association study reported genetic variants related to the fungal α-diversity and to the abundance of *Blastocystis* spp. Annotated candidate genes were mainly associated with immunity, gut homeostasis and metabolic processes. Additionally, we explored the association of gut commensal eukaryotes with piglet body weight. Our results pointed to a positive contribution of fungi from the *Kazachstania* genus, while protists displayed both positive (*Blastocystis* and *Entamoeba*) and negative (*Trichomitus*) associations with piglet body weight.

**Conclusions:**

Our results point towards a minor and taxa specific genetic control over the diversity and composition of the pig gut eukaryotic communities. Moreover, we provide evidences of the associations between piglets’ body weight after weaning and members from the gut fungal and protist eukaryote community. Overall, this study highlights the relevance of considering, along with that of bacteria, the contribution of the gut eukaryote communities to better understand host-microbiome association and their role on pig performance, welfare and health.

## Introduction

The gut microbiome harbors thousands of species of archaea, bacteria, viruses and eukaryotes such as protists and fungi that contribute to host biology. The gut eukaryotic communities of monogastric species show a higher interindividual variability, but less abundance, diversity and richness than their bacterial counterparts [1]. The so called gut mycobiome of healthy humans is dominated by fungal genera like *Saccharomyces*, *Candida, Penicillim, Aspergillus* and *Malassezia* [2], while protist genera such as *Blastocystis, Entamoeba* and *Enteromonas* have been reported in the human gut across different geographical location [3–5]. There is currently considerable interest in understanding the mechanisms through which gut commensal eukaryotic communities contribute to host homeostasis and health [6]. The role of fungi and protists in the human gut remains poorly understood, but some authors suggest that commensal species may induce innate immune response in the host [7], and they could also have other potential benefits [5, 8, 9]. For example, *Blastocystis* spp. and non-pathogenic *Entamoeba spp*. have been associated with a healthy and highly diverse gut microbiome ecosystem [6, 10, 11], while *Tritrichomonas musculis* modulates the intestinal immune system and increases host protection against mucosal bacterial infections in mice [12].

The few studies about gut eukaryotes conducted in pigs (*Sus scrofa domesticus*) used a limited sample size and mainly focused on isolated members of the eukaryote communities or parasite identification under pathogenic conditions [13–18]. In swine, *Kazachstania* spp. and members of the *Saccharomycetaceae* family are predominant in the gut mycobiota [13–16], while gut protist community of healthy pigs is dominated by *Blastocystis* spp., *Tritrichomonas* spp. and *Balantidium coli*. Environmental factors such as diet seems to play a key role in modulating the structure and composition of both eukaryote and prokaryote communities. However, little is known about the genetic control of the gut eukaryotes, since published studies have been focused on the role of host genetics in shaping the gut bacterial communities [19–21].

The main goal of this study is to characterize commensal fungi and protists inhabiting the gut of healthy pigs, explore the putative host genetic control over diversity and composition of gut eukaryotes communities, and analyze their association with piglets body weight.

## Results

### Composition of the pig gut microbial eukaryotic communities

**Fig. 1:**
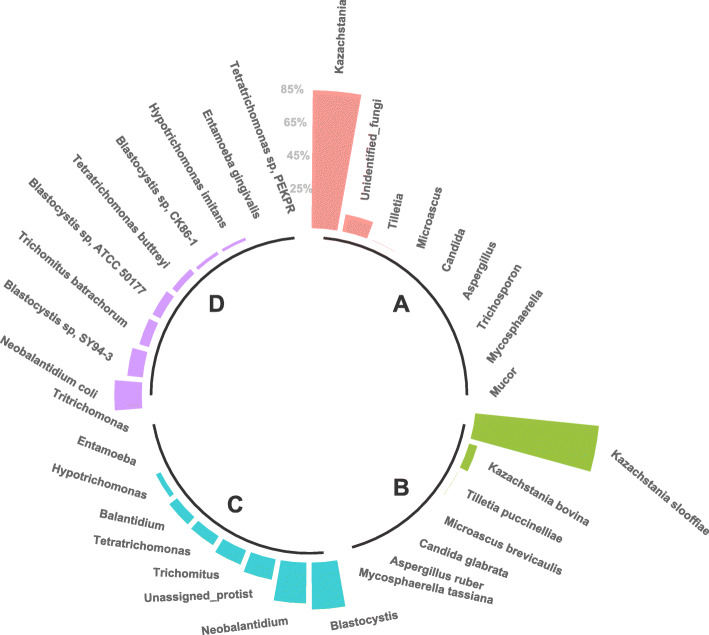
Circular barplot representing the relative abundance of the most abundant fungal Genus (**a**) and species (**b**) as well as protists Genus (**c**) and species (**d**)

The pig gut microbial eukaryote communities from 514 healthy pigs were analyzed by sequencing the 18S rRNA gene and the ITS2 region. After quality control, 492 fungal and 227 protist ASVs were identified. The pig gut mycobiota was dominated by yeast from the *Kazachstania* genus, in particular by the species *K. slooffiae* and *K. bovina* (Fig. 1). Other yeasts associated to the *Candida* genus were identified, such as *C. glabrata*, *C. albicans* and *C. (Diutinia) catenulata*, which were present in 2–7% of the animals (Additional file 1 Table S1). Besides, a moderate prevalence (15% of pigs) of *Tilletia puccinelliae* was also observed (Additional file 1 Table S1). Along with the ascomycete *Saccharomyces arboricola*, a number of basidiomycetous yeasts were also detected with relatively low incidence (< 2%), such as *Sporobolomyces roseus*, *Trichosporon dohaense*, *Debaryomyces prosopidis*, *Pichia sporocuriosa*, *Filobasidium globisporum* and *Vishniacozyma victoriae*. Finally, the pig intestinal mycobiota included a third and more diverse group of cosmopolitan fungal species that are generally categorized as “soil fungi”. In most cases they have a relatively low occurrence in gut (< 2%) and are primarily associated with a general saprotrophic ecophisiology (i.e. *Aspergillus* spp., *Aureobasidium pullulans*, *Cladosporium tenuissimum*, *Mucor circinelloides* and *Penicillium polonicum*).

With regard to protists, the prevalent ASV corresponded to an unidentified species of the subclass *Trichostomatia* (superphylum *Alveolata* in the SAR supergroup). Furthermore, the species *Neobalantidium coli* from this class was found in 82% of the animals (Additional file 1 Table S1). Likewise, with a relatively high incidence and average abundance (up to 99 and 29%, respectively), a number of *Blastocystis* spp. (superphylum *Stramenopiles* in the SAR supergroup). Other detected commensal trichomonads included *Tetratrichomonas buttreyi* (94% incidence), *Hypotrichomonas imitans* (67% incidence), as well as the *Tetratrichomonas* strain PEKPR (10% incidence), and with a very low incidence and abundance (< 1%), *Tritrichomonas suis*. Finally, one representative of the ameboid protist species *Entamoeba gingivalis* (supergroup *Amoebozoa*) was also detected with a relatively high incidence (77%) but a low abundance (< 1%).

### Host and environmental factors modulating the diversity of pig gut microbial eukaryotes

The modulatory effect of host and environmental factors over the diversity and composition of gut eukaryotic communities in pigs was evaluated with different approaches. Results from the first PERMANOVA with the whole dataset indicated that the combination of farm and animal age represented the most significant effect shaping the gut eukaryotic communities (*p* < 0.0001), explaining around 44 and 42% of the total variability of fungal and protist communities, respectively. The same data structure was recovered by the principal coordinates analysis (PCoA) based on the Bray–Curtis distance, which showed two clusters that match with sample farms origin (Additional file 2 Figure S1). The diversity index also revealed important differences between farms/ages (Kruskal–Wallis test). Samples taken at 190 days of age in the experimental farm showed a significantly higher protist (*p* = 0.013) and fungal (*p* = 0.006) alpha-diversity (Additional file 2 Figure S1). In contrast, weaned piglets raised in commercial conditions showed higher protist beta-diversity. A second PERMANOVA within farm/age was performed. Results of weaned piglets in the commercial farm indicated an important effect of the batch (*p*-value< 0.0001) on alpha-diversity of both fungal and protist communities, whereas the pen effect in the second dataset (experimental farm) seems to affect exclusively protist diversity (Table 1). Regarding sex effects, gut fungi alpha-diversity differed between sexes in weaned piglets (p-value = 0.02), whereas no differences between castrated males and females mycobiota diversity were observed at 190 d of age (Table 1).

**Table 1:** Resume of the permutational multivariate analysis of variance

Dataset	Community	Bacht R2 (P-value)	Sex R2 (***P***-value)
Weaned piglets (*n* = 405) commercial farm	Fungi	0.12 (*p* < 0.001)	0.01 (*p* = 0.02)
	Protist	0.07 (*p* < 0.001)	0.02 (*p* = 0.60)
Finishing pigs (*n* = 109) experimental farm	Fungi	0.09 (*p* = 0.35)	0.01 (*p* = 0.24)
	Protist	0.12 (*p* = 0.03)	0.01 (*p* = 0.55)

Afterwards, we assessed the degree of host genetic control over the diversity and composition of gut protist and fungal communities by estimating their heritabilities. ﻿The estimated heritability of the α-diversity index was low, with posterior mean estimates of ranging from 0.161 to 0.188 for fungi and protists, respectively (Table 2). The posterior highest density regions at 95% encompassed values between 0.067 and 0.299, indicating a probably very limited but not negligible heritability for these traits. In the same way, the heritabilities of most representative fungal and protist genera abundance reached low to moderate (Table 2). The abundance of the protist *Blastocystis* genus was the most heritable (posterior mean 0.281, SD 0.101), followed by the abundance of *Trichomitus* (0.273, SD 0.090) and *Neobalantidium* (0.233, SD 0.077) genera. Also, a heritability around 0.209 was estimated for the abundance of the preponderant fungal genus *Kazachstania*, and lightly larger (0.216) for the abundance of *Kazachstania slooffiae* species.

**Table 2:** Mean (standard deviation) and highest density regions at 95% of marginal posterior distributions for heritabilities of pig gut eukaryotes microbial traits

Community	Level	Trait	Heritabiliy (SD)	HDR
Fungi	α-diversity	Shannon-index	0.161 (0.053)	0.067–0.266
	Genus	*Kazachstania*	0.209 (0.064)	0.095–0.333
	Specie	Kazachstania_slooffiae	0.216 (0.071)	0.093–0.353
Protist	Alpha-diversity	Shannon-index	0.188 (0.060)	0.077–0.299
	Genus	*Blastocystis*	0.281 (0.101)	0.096–0.483
		*Trichomitus*	0.233 (0.077)	0.085–0.383
		*Tetratrichomonas*	0.159 (0.051)	0.070–0.258
		*Neobalantidium*	0.273 (0.090)	0.108–0.443
	Species	Trichomitus batrachorum	0.240 (0.075)	0.100–0.382
		Tetratrichomonas buttrey	0.158 (0.048)	0.070–0.256
		Neobalantidium coli	0.256 (0.086)	0.100–0.425
		Blastocystis sp. ATCC 50177	0.245 (0.086)	0.086–0.409
		Blastocystis sp. CK86–1	0.203 (0.066)	0.081–0.325
		Blastocystis sp. SY94–3	0.205 (0.065)	0.085–0.334

### Identification of host genetic regions linked with the composition and diversity of pig gut microbial eukaryotes

**Fig. 2:**
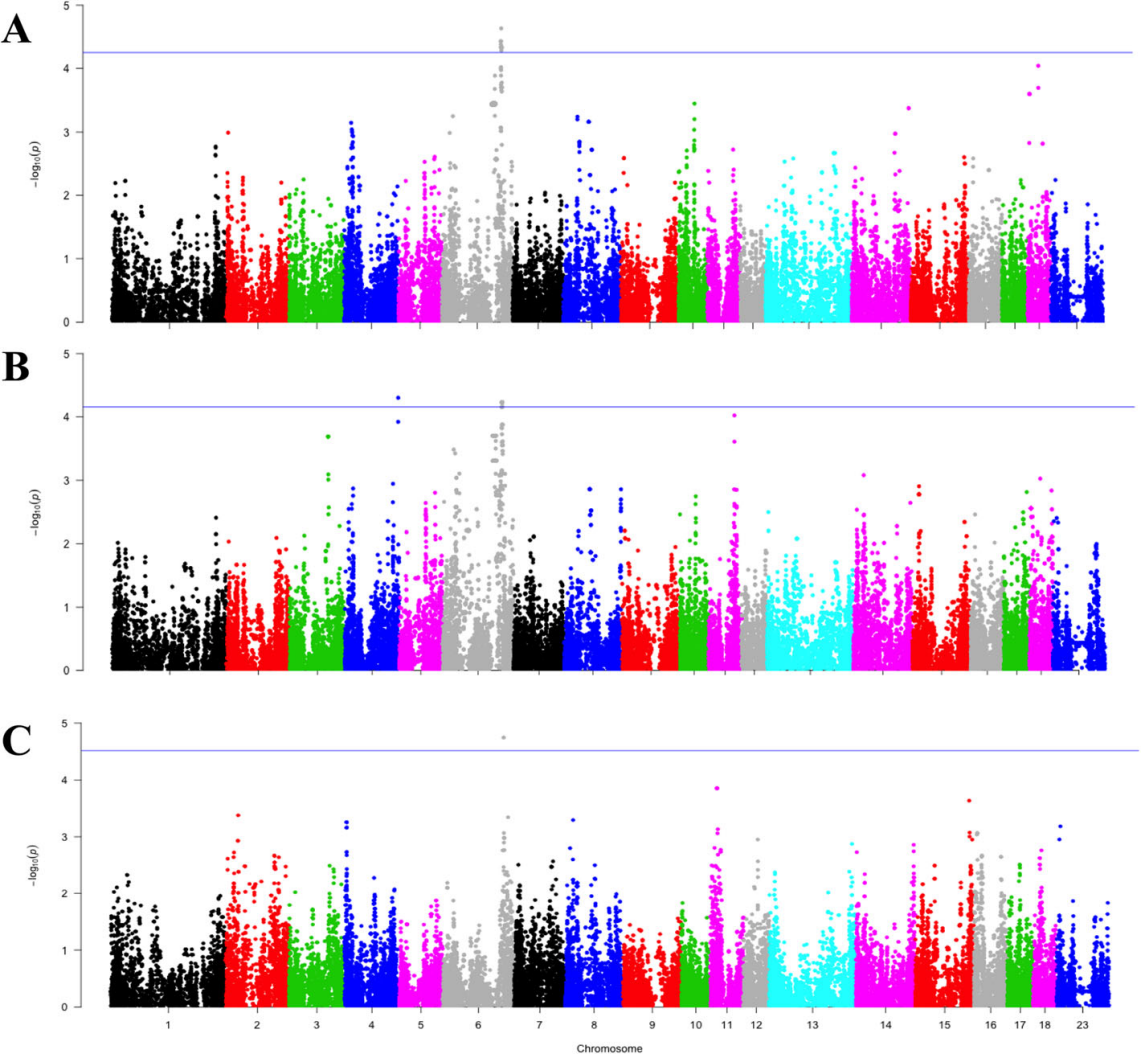
Significance across the whole genome of the association between SNP markers and the relative abundance of (**a**) Blastocystis sp. ATCC 50177, (**b**) Blastocystis sp. CK8166 and (**c**) Fungi Alpha diversity. The x-axis represents the pig autosomal chromosomes (1–18), and the y-axis the significance level represented as the –log_10_ (*P*-value)

Results from GWAS revealed few and weak association signals between the host genome and the gut microbial eukaryotes composition and diversity. We identified a total of 174 SNPs as significantly (at chromosome-wide level, FDR < 0.05) related with the abundance of protist community (Additional file 3 Table S2), located in seven intervals distributed across three pig *Sus scrofa* chromosomes: SSC6, SSC17 and SSC18 (Table 3). The 32.18% of these SNPs were intronic variants, 56.89% were intergenic, 5.75% were located upstream/downstream of genes, 1.15% were exonic synonymous, and 4% mapped within non-coding transcript variants (Additional file 3 Table S2). Most of these associated SNPs (164 out of 174) were identified on SSC6 (Table 3), and were mainly associated with the relative abundance of two species of *Blastocystis* genera: *CK86–1* and *ATCC 50177* (Fig. 2). The remaining significant SNPs located on SSC17 (46.77–46.99 Mb interval) and SSC18 (two intervals: 4,53–4,57 Mb and 25,85–25,88 Mb) were also associated to the relative abundance of members of *Blastocystis* (Table 3). Regarding diversity, the aforementioned 141.91–145.39 Mb region of SSC6 resulted also associated with the fungi Shannon diversity-index (Fig. 2). Finally, no significant associations with either the relative abundance of fungi (*Kazachstania* genera or *Kazachstania slooffiae*) or the diversity of protist were observed.

**Table 3:** Resume of the chromosomal intervals identified by the GWAS

Chr	Start (bp)	End (bp)	Size (Mb)	Associated microbial trait
6	68,999,679	69,466,937	0.467	*Blastocystis* sp. SY94–3
6	120,437,893	121,498,044	1.060	*Blastocystis* sp. ATCC 50177; *Blastocystis* sp. CK86–1
6	122,778,463	132,592,988	9.814	*Blastocystis* sp. ATCC 50177; *Blastocystis* sp. CK86–1
6	141,914,558	145,392,721	3.478	*Blastocystis* sp. ATCC 50177; *Blastocystis* sp. CK86–1; ITS α-diversity
17	46,764,111	46,988,559	0.224	*Blastocystis* sp.
18	4,538,602	4,570,818	0.032	*Blastocystis* sp. ATCC 50177
18	25,850,358	25,885,196	0.035	*Blastocystis* sp. ATCC 50177

### Genes and pathways associated to pig gut microbial eukaryotes

A total of 229 protein-coding, 74 long non-coding RNA and two miRNAs (*mir138–2* and *ssc-mir-186*) (Additional file 4 Table S3) were annotated within the host genomic intervals associated to the gut microbial eukaryotes composition and diversity. A detailed exploration of annotated genes revealed that the Interleukin 23 Receptor gene (*IL23R*) holds two intronic SNPs, rs343576943 (*p*-value = 1,8*10^− 5^) and rs81392455 (p-value = 1,8*10^− 5^) that were associated with fungal α-diversity. Furthermore, the variants rs81391297, rs81391299 and rs81299019, mapped within the 122.8–132.6 Mb interval on SSC6 and associated with the relative abundance of *Blastocystis* spp., were located on intron eight of the Phosphatidylinositol 3-Kinase Catalytic Subunit Type 3 (*PIK3C3*) gene. On SSC17, the intronic variant rs331945396 (SSC17:46832698–46,832,698) in the Hepatocyte Nuclear Factor 4 Alpha (*HNF4A*) gene was also associated with the relative abundance of *Blastocystis* genera (p-value = 9,02*10^− 5^). Other genes related to the immune system were annotated within the aforementioned chromosomal intervals, as for example the TNF Receptor Superfamily Member 9 (*TNFRSF9*), the Interleukin 12 Receptor Subunit Beta 2 (*IL12RB2*) or the Phosphatidylinositol-4,5-Bisphosphate 3-Kinase Catalytic Subunit Delta (*PIK3CD*), as well as other members of immune-related pathways (e.g. *CDH1*, *CDH11*, *CDH3*, *CDH5*, *LPAR3*, *PIK3C3*, *CCL17*, *CCL22*, *CDH5*, *CKLF* and *MMP15*). Finally, the functional annotation revealed that these genes belong to a variety of physiological processes and gene networks related to Cell-To-Cell Signaling and Interaction, Cellular Assembly and Organization, Cellular Function and Maintenance, Developmental Disorder, Hereditary Disorder, Cell-mediated Immune Response, Humoral Immune Response, Immune Cell Trafficking, and Protein Synthesis (Additional file 5 Table S4). Metabolic pathways most significantly enriched by the list of candidate genes include Relaxin Signaling, Leptin Signaling in Obesity, IGF-1 Signaling, PXR/RXR Activation and HIF1α Signaling pathways. It should be noted the overrepresentation of immune-related pathways such as Gα12/13 Signaling, IL-9 Signaling, IL-23 Signaling, IL-12 Signaling, ERK/MAPK Signaling, Agranulocyte and Granulocyte Adhesion and Diapedesis (Additional file 6 Table S5).

### Association between pig gut eukaryotes and host performance

**Fig. 3:**
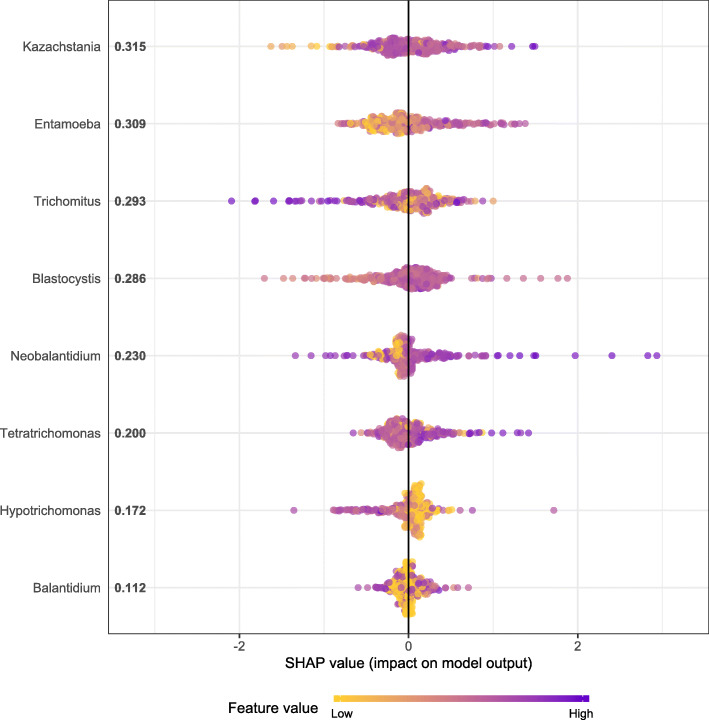
Relevance of fungal and protist genera regarding piglets body weight, according to the Shapley Additive Explanation (SHAP) values. The x-axis represents the SHAP value, the y-axis shows the genera names in order of importance from top to bottom, and the value correspond to the genera mean SHAP value

Most relevant fungal and protist genus associated with body weight are summarized in Fig. 3. The result suggested association between piglets’ body weight and the abundance of *Kazachstania* fungal genera as well as protist members of *Entamoeba, Trichomitus, Tetratrichomonas* and *Blastocystis*. The Shapley Additive Explanation (SHAP) values showed a nonlinear association between *Kazachstania* and *Blastocystis* abundance and piglets body weight (Additional file 7 Figure S2). Furthermore, *Entamoeba* on one side, and *Trichomitus* on the other, were positive and negatively associated with body weight (Additional file 7 Figure S2).

## Discussion

The analysis of gut microbial eukaryotic communities in 514 healthy animals have allowed characterizing commensal fungi and protists inhabiting the porcine gut tract. In agreement with previous reports in pigs [22–24] our results evidenced that pigs gut mycobiota is dominated by the yeast *Kazachstania* spp. which corresponds to the teleomorphic state of *Candida slooffiae* and *Candida bovina* [25]. Particularly the species *K. slooffiae* was found in all studied animals across farms, sex and ages, observation that is in agreement with the ubiquitous detection of this yeast from the gut of healthy pigs, and further supports the hypothesis that pigs intestinal tract is the primary ecological niche for *K. slooffiae* [13]. The closely related *K. bovina* was detected in the 45% of the studied pigs, and might also play a significant role in the gut. Several transient fungi that probably arise from the feeding or the environment were observed but with low incidence. These basidiomycetous yeasts have often been isolated from the phyllosphere [26] and are also known to cause human opportunistic infections in certain cases [27]. The plant-gut association for certain yeast is more evident in a second group of filamentous fungi that are characterized as specialized plant biotrophs. The most representative of those is the basidiomycete *T. puccinelliae* that, surprisingly, occurred in 15% of the studied pigs. Species from *Tilletia* genera are smut fungi that infect various grasses from the *Poaceae* family and encompass plant pathogens of economic importance in the production of cereals and forage grasses. So far, *T. puccinelliae* has only been isolated from weeping alkaligrass (*Puccinellia distans*), a common ruderal grass in Europe and North America [28]. Plants infected by other *Tilletia* species do not pose a toxicity risk for humans but contaminated grains might be derived to animal feed due to off-flavors [29]. Such association between feed and fungal gut might also apply for the plant pathogens *Ustilago hordei* and *Mycosphaerella tassiana*, found in about 3% of the animals. Former results are in agreement with previous reports on humans and non-human primates [2, 30–32], and suggest that, differently to pig gut prokaryotes [33], the pig gut mycobiome may lack a stable core. Consequently, it is expected that a large proportion of the fungi detected in pig fecal samples may be transients from dietary or environmental origin. Meanwhile, the prevalence (> 80% of the 514 pig samples) of four protist genera (*Blastocystis, Neobalantidium*, *Tetratrichomonas* and *Trichomitus*) was observed, which is in agreement with previous reports in pigs [17, 34]. *Neobalantidium* is a world-wide parasitic-opportunistic human pathogen that is transmitted through the fecal-oral route, particularly when there is a close contact with pigs, which are its asymptomatic reservoir hosts [35]. *Blastocystis* is a ubiquitous protozoan of human and pigs, commonly found in healthy populations [6, 36]. However, several species in *Blastocystis* are causative agents of diarrhea in humans through the fecal-oral infection route, being the pig a common reservoir [37]. A number of protists in the *Trichomonadida* order (supergroup *Excavata*) that are common in the digestive tract of pigs [38] were also found in the present study. Colonization with this species has not been associated to virulence in mammals, but the full pathogenic potential of *T. batrachorum* has yet to be explored.

The analysis of host intrinsic and extrinsic factors ascertained the significant modulatory role of the environmental effects gathered in batch and farm factors (e.g. climate, management conditions, diet) on gut eukaryotes composition and diversity. Also important differences between the diversity of gut eukaryotic communities in weaned and finishing pigs were evidenced, whereas scarce differences due to the sex of the animal were observed. We explored the putative host genetic control of the diversity and composition of both gut protist and fungal communities. Despite the relatively low heritability estimates (posterior means from 0.158 to 0.281), they represent the first reported evidence of certain host genetic determinism of gut eukaryotic profile in pigs. The abundance of the protist *Blastocystis* genus was the most heritable, with around 28% of their variability explained by the genetic variability of the host, followed by the abundance of *Trichomitus* and *Neobalantidium* genus, and the preponderant fungal genus *Kazachstania*. Lower but not negligible heritabilities were obtained for a particularly complex trait such as the α-diversity index in both protist and fungi, which allows hypothesizing that pigs can have a limited genetic tendency to harbor more or less diverse eukaryotic communities in its gut. These results should be taken with caution because of the limited sample size, but as a whole they allow inferring a limited and taxa specific genetic control over diversity and composition of gut eukaryotes. Besides, GWAS revealed genetic variants associated at chromosomal level with fungal α-diversity (on SSC6) and the abundance of *Blastocystis* spp. (on SSC6, 17 and 18). Two intronic SNPs associated with fungal α-diversity were mapped on the *IL23R* gene. Remarkably, this gene, which is highly expressed in Th17 cells, seems to play an important role in the proliferation and survival of these cells, which are critical for host defense against fungal infections [39]. In fact, genetic variants in the *IL23R* gene have been associated with different susceptibility to fungal infections [40] and *IL-23R* deficient mouse was susceptible to systemic infection with *C. albicans* [41]. Furthermore, IL-23R had been reported to be involved in the diversity of ileum microbiota in humans [42]. In the same chromosome, *PIK3C3* gene gathered two SNPs associated to *Blastocystis* abundance. *PIK3C3* is required for T cell homeostasis [43–45] and reported to play a relevant role in maintaining gut homeostasis [46]. Other genes involved in the intestinal epithelial integrity and gut homeostasis were annotated on SSC17, such as *HNF4A*, a relevant regulator that mediates microbial control of intestinal gene expression [47]. Also a number of genes related to the immune system were annotated within the aforementioned chromosomal intervals, as the *TNFRSF9* gene that contributes to the development, survival and activation of T cells [48, 49], the *IL12RB2* and *PIK3CD* genes, and a plethora of members of the immune-related pathways Gα12/13 Signaling, IL-9 Signaling, IL-23 Signaling, IL-12 Signaling, ERK/MAPK Signaling, Agranulocyte and Granulocyte Adhesion and Diapedesis. Several studies, most of them performed in cell lines, have tried to shed light on *Blastocystis*–host interactions. These studies have shown that *Blastocystis* are able to disrupt intestinal barrier integrity and function, and that they modulate the host immune response through the degradation of IgA, the suppression of iNOS, and the induction of pro-inflammatory cytokines such as IL-12 and IL-23, among others [50, 51]. Furthermore, *Blastocystis* subtype-dependent upregulation of pro-inflammatory cytokines in macrophages through the activation of MAPK pathways has been described [52]. Overall, it is noteworthy to highlight that in our study genes related to pathways previously associated with *Blastocystis* experimental infections were identified as being associated with *Blastocystis* relative abundance. Although further genetic and functional analyses are needed to better understand how the biological mechanisms of the host could modulate the diversity and abundance of fungi and protists, and their relationship with the gut bacterial counterparts. These findings are in line with recent reports in mice [53], and suggest that polymorphisms located in (or in linkage disequilibrium with) genes related to immune system and gut homeostasis may modulate the diversity and composition of the eukaryote gut communities in pigs. The evolutionary mechanism beyond the observed association may be explained by indirect relationships between mutual favorable selection at host-genome and microbial level [54, 55]. Although far to be fully understood, some examples of host-genetic microbiota associations have been reported (reviewed in [55]), that similar to our results identify candidate genes mainly related to host-response against pathogens, sensing microbes, cell signaling pathways and innate immune system. However, in agreement with previous reports centered in gut bacterial [53, 56], our findings also indicate a limited and target specific host genetic control over the composition of the gut eukaryote communities in pig.

On another level, we provided evidences of the association between piglets body weight after weaning and members from the fungal and protists gut eukaryote community. A previous study with growing piglets, reported no significant effects on growth performance parameters upon oral supplementation with *K. slooffiae* [57]. However, a recent detailed metabolic study conducted by the same authors [58] under laboratory conditions with cultures of *K. slooffiae*, concluded that this yeast produces peptides and short chain fatty acids that might benefit the gut health, and provides an additional protein source that contains essential amino acids and other useful growth factors for the animal [58]. Consequently, it seems plausible that the observed relationships between *Kazachstania* and piglets body weight partially relies on the *K. slooffiae* positive effect on gut health through the production of essential amino acids and short fatty acids that are absorbed by the host or employed by others microbial members of the pig gut microbiome. On the other hand, the impact of members of the gut eukaryotic community to growth performance and the digestive enzyme activities have been reported in shrimp [59]. Furthermore, *Blastocystis spp*. and non-pathogenic *Entamoeba spp*. have been associated with a healthy and diverse gut microbiome ecosystem [6, 10, 11]. Likewise, mechanisms of horizontal gene transfer of genes associated with cellulose degradation and carbohydrate use from bacterial to protist such as *Blastocystis* have been documented [60–62]. Therefore, we hypothesize that observed positive relationships between piglets body weight at 60 days and members of *Blastocystis* and *Entamoeba* may be explained by the capacity of *Blastocystis* to degrade carbohydrates, which are routinely included after weaning in a typical transition diet. An alternative explanation may be also the recognized [6, 10, 11, 62] positive effects of *Blastocystis* and *Entamoeba* over the microbiota diversity and richness, which is turn is associated with host gut health.

In summary, our findings highlight the relevance of considering the gut eukaryotic communities to better understand the porcine gastrointestinal microbiome and its contributions to host performance and health. At that point we cannot neglect the contribution of other genera that might be relevant at different ages, under different environmental conditions of for other phenotypic traits. We are also aware of some limitations of our study, as for example the limited sample size or the lack of degrees of freedom to estimate all effects, but also of methodological constraints such as primers choice or the use of less curated (compared to bacterial) protists and fungal databases, that together with the reduced amount of public available reference genomes compromise the accuracy of the taxonomic classification. To the best of our knowledge, this study represents the largest effort to characterize the gut fungal and commensal protist communities in pigs, but further larger studies including experimental validations and alternative meta-sequencing approaches are needed to unveil the role of the host-associated microbial communities in pigs production performance, welfare and health.

## Conclusions

The diversity and composition of gut commensal eukaryotic communities in healthy pigs at two ages have been characterized. The porcine gut mycobiota is dominated by yeast, with a high prevalence of *Kazachstania*, and a common set of four protist genera (*Blastocystis, Neobalantidium, Tetratrichomonas and Trichomitus*) persisted through the majority of animals. Our results point towards a minor and taxa specific genetic control over the diversity and composition of the pig gut eukaryotic communities, but we describe associations with genes functionally related to the immune system and gut homeostasis that might have an effect in modulating the fungi α-diversity and the abundance of *Blastocystis* ssp. We provide also evidences of the associations between piglets body weight after weaning and members from the gut fungal and protist eukaryote community. Overall, our findings highlight the relevance of considering, together with that of bacteria, the contribution of gut eukaryotic communities to better understand the host-microbiome association and its role on pig performance, welfare and health.

## Methods

### Sample collection, DNA extraction and sequencing

Fecal samples were collected at two ages from 514 healthy Duroc pigs belonging to the same commercial outbred line but allocated in two different farms. A total of 405 weaned piglets (204 males and 201 females) distributed in seven batches were sampled in a commercial farm at 60 ± 8 days of age and mean body weight around 18.58 kg (SD 3.13), after 4 weeks receiving the same transition-based diet (Additional file 8 Table S6). The remaining 109 pigs (50 castrated males and 59 females) were raised under intensive standard conditions at IRTA experimental farm (IRTA, Monells, Spain), and fecal samples were collected at 190 ± 5 days of age, when they fed a finishing standard diet. Both groups of pigs were genetically connected. Animal care and experimental procedures were carried out following national and institutional guidelines for the Good Experimental Practices and were approved by the IRTA Ethical Committee.

DNA was extracted with the DNeasy PowerSoil Kit (QIAGEN, Hilden, Germany), following manufacturer’s instructions. Extracted DNA was sent to the University of Illinois Keck Center for Fluidigm sample preparation and sequencing. The Internal Transcribed Spacer 2 (ITS2) region was amplified using primers ITS3: 5′-GCATCGATGAAGAACGCAGC-3′ and ITS4: 5′-TCCTCCGCTTATTGATATGC-3′ [63]. Protist-specific primers [64], F-566: 5′-CAGCAGCCGCGGTAATTCC-3′ and R-1200: 5′-CCCGTGTTGAGTCAAATTAAGC-3′ were used to amplify the 18S rRNA gene fragment. Amplicons were paired-end (2 × 250 nt) sequenced on an Illumina NovaSeq (Illumina, San Diego, CA, USA).

### Bioinformatics and statistical analysis

Sequences were analysed with *Qiime2* [65], barcode sequences, primers and low-quality reads (Phred scores of < 30) were removed. The quality control also trimmed sequences based on expected amplicon length and removed chimeras. Afterwards, sequences were processed into Amplicon Sequences Variants (ASVs) at 99% of identity. ASVs present in less than three samples and representing less than 0.005% of the total counts were filtered out. Samples with less than 6000 (fungi, *n* = 21 samples) or 10,000 (protists) reads were also excluded. ASVs were classified to the lowest possible taxonomic level based on SILVA v123 database [66] for 18S rRNA genes, and the UNITE QIIME version (release 18.11.2018) for fungi [67]. Subsequently, we excluded those ASVs not taxonomically classified as protists or fungi. Moreover, we verified the fungi taxonomic assignation following the recommendation of Nilsson [68] by a manual examination of the most abundant fungal ASVs against the International Nucleotide Sequence Database (http://www.insdc.org/). Before the estimation of diversity indexes, samples were rarefied at 6000 (fungi) and 10,000 (protists) reads of depth, to allow an equal depth. Diversity metrics were estimated with vegan R package [69]. Alpha-diversity was evaluated with the Shannon index [70], and Beta-diversity was assessed using the Whittaker index [71]. To identify environmental (farm, batch or pen) and host-covariates (sex, age, body weight) that may modulate the diversity, structure and profile of the eukaryote communities, we run a Permutational multivariate analysis of variance (PERMANOVA) test using the *adonis* function from vegan [69]. Significance levels were determined after 10,000 permutations and the multiple comparison tests were performed using False Discovery Rate (FDR).

### Genotype data and estimation of heritability

The putative host genetics determinism of gut eukaryotic profile in pigs was initially assessed by estimating the heritability (*h*^2^) of both fungi and protists alpha-diversity, as well as of their taxa abundance. For these analyses, we used the samples taken at 60 ± 8 days of age in the commercial farm. The Porcine 70 k GGP Porcine HD Array (Illumina, San Diego, CA) was used to genotype 390 out of 405 animals sampled in the commercial farm. Parameters estimation was performed using the following Bayesian mixed model implemented with the *bglr* R package [72]:

$$ {y}_{ijk}={sex}_j+{b}_k+{u}_i+{e}_{ijk} $$

where *y*_*ijk*_ corresponds to the alpha-diversity (fungi or protist) or taxa (genera or specie) clr-transformed abundance of the 푖^th^ individual of sex *j* in the *k*^th^ batch; *sex*_*j*_ and *b*_*k*_ correspond to the systematic effects of *j*^th^ sex (2 levels) and *k*^th^ batch effect (7 levels), respectively_;_*u*_i_ is the random genetic effect of individual *i*, distributed as case **퐮**∼푁(0,**퐆**휎^2^_푢_) being **퐆** the genomic relationship matrix calculated using the filtered autosomal SNPs based on the methodology of Yang et al. [73]; finally, *e*_*ijk*_ is the random residual term, with a distribution **e**∼푁(0,**I**${\sigma}_e^2$). The model was run using a Gibbs sampler with 30,000 iterations and a burn-in of 2000 rounds. Posterior sample mean and standard deviation of the heritability $\left({h}^2={\sigma}_u^2/\left({\sigma}_u^2+{\sigma}_e^2\right)\right)$ were obtained from the resulted posterior distributions.

### Genome wide association study (GWAS)

Quality control was performed with plink [74] to exclude single nucleotide polymorphisms (SNPs) with minor allele frequencies < 5%, rates of missing genotypes above 10%, as well as SNPs that did not map to the porcine reference genome (Sscrofa11.1 assembly). Then, to identify SNPs from the host genome associated with the alpha-diversity as well as protists and fungi relative abundances, genome-wide association studies (GWAS) were performed between 42,608 SNPs and the alpha-diversity or the centered log ratio (clr) transformed genera and species abundance tables. Only the genera and species fully taxonomically classified and present in more than 80% of the samples were included in the analysis. For this propose, the genome-wide complex trait analysis (GCTA) software [73] was employed using the following model at each SNP:

$$ {y}_{ijk}={sex}_j+{b}_k+{u}_i+{s}_{li}{a}_l+{e}_{ijk} $$

where *y*_*ijk*_ corresponds to the alpha-diversity (fungi or protist) or taxa (genera or specie) clr-transformed abundance of the 푖^th^ individual of sex *j* in the k^th^ batch; *sex*_*j*_, *b*_*k*_ and *u*_*i*_ are, respectively, the effects of sex, batch and infinitesimal genetic effect described in the previous model; *s*_*li*_ is the genotype (coded as 0,1,2) for the *l*^th^ SNP of individual *i*, and *a*_*l*_ is the allele substitution effect of SNP *l* on the analysed trait. A SNP was considered to be significantly associated if the corresponding *p*-value was lower than 0.05, after Benjamini–Hochberg [75] correction for multiple testing at chromosome level.

### Gene functional classification and canonical pathway analyses

Functional classification and pathway analyses of the annotated candidate genes were carried out using the Ingenuity Pathways Analysis software (IPA; Ingenuity Systems, http://www.ingenuity.com). Significance levels for enrichment of each canonical pathway in the list of candidate genes were calculated using Fisher’s exact test, and the resulting *p*-values were corrected for multiple-test using the Benjamini and Hochberg algorithm [75]. The cut-off for considering an enrichment as significant was established at a corrected p-value < 0.05.

### Association between pig gut eukaryote communities and body weight

The associations between fungal and protist genera abundance and piglest body weight at 60-days was evaluated using the dataset from the commercial farm (*n* = 405). In a first step we implemented XGBoost, a fast algorithm which incorporates a penalization term in the loss function to prevent overfitting (Chen & Guestrin, 2016) using the XGBoost R packages. Afterwards, the importance of the variables was evaluated with Shapley Additive Explanation (SHAP) algorithm. SHAP (Lundberg & Lee, 2017) is an extension of the coalition game from the game theory (Shapley, 1953), which is used in a context of multicollinearity. Basically, the model evaluates the importance of each variable by measuring a conditional contribution, i.e., the effect caused in the prediction by including that variable. SHAP values were obtained and visualized using the ShapXGboost R package [76].

## Supplementary information

**Additional file 1 Table S1.** Description of the prevalence and relative abundance of the pig gut eukaryotes communities.

**Additional file 2 Figure S1.** Sample distribution of fungi (A) and protist (B) communities. Diversity indexes of fungi (C) and protist (D) communities. Blue color represents the finishing pigs (experimental farm at 190 days) and red the weaned piglets (commercial farm at 60 days).

**Additional file 3 Table S2.** Description of the genetic markers identified as significantly according to the genome-wide association studies.

**Additional file 4 Table S3.** Description of the genes annotated within the genomic intervals.

**Additional file 5 Table S4.** Functional annotation of the identified candidate genes.

**Additional file 6 Table S5.** Metabolic pathways significantly enriched by the list of candidate genes.

**Additional file 7 Figure S2.** Patterns of association between the piglets body weight at 60-days old and the fungal and protist abundance according to the Shapley Additive Explanation (SHAP) values. The x-axis represent the genera abundance after Cumulative Sum Scaling (CSS) normalization and log transformation. Y-axis represents the SHAP values of the piglets’ body weight.

**Additional file 8 Table S6.** Description of the ingredients and nutrient content of the diets used during the experiment.

## Data Availability

The raw sequencing data employed in this article has been submitted to the NCBI’s sequence read archive (https://www.ncbi.nlm.nih.gov/sra); BioProject: PRJNA608629.

## Abbreviations

QIIME: Quantitative insights into microbial ecology
ITS: Internal transcribed spacer
ASV: Amplicon sequence variant
clr: Centered log ratio transformation
GWAS: Genome-wide association studies
XGBoost: eXtreme Gradient Boosting
FDR: False discovery rate
