# Supplementary material for: Gut eukaryotic communities in pigs: diversity, composition and host genetics contribution
Source: Anim Microbiome. 2020 May 7;2:18. doi: 10.1186/s42523-020-00038-4 (PMC7807704; doi:10.1186/s42523-020-00038-4)

Supplementary figure 1. Sample distribution of fungi (A) and protist (B) communities. Diversity indexes of fungi (C) and protist (D) communities. Blue color represents the finishing pigs (experimental farm) and red the weaned piglets (commercial farm).

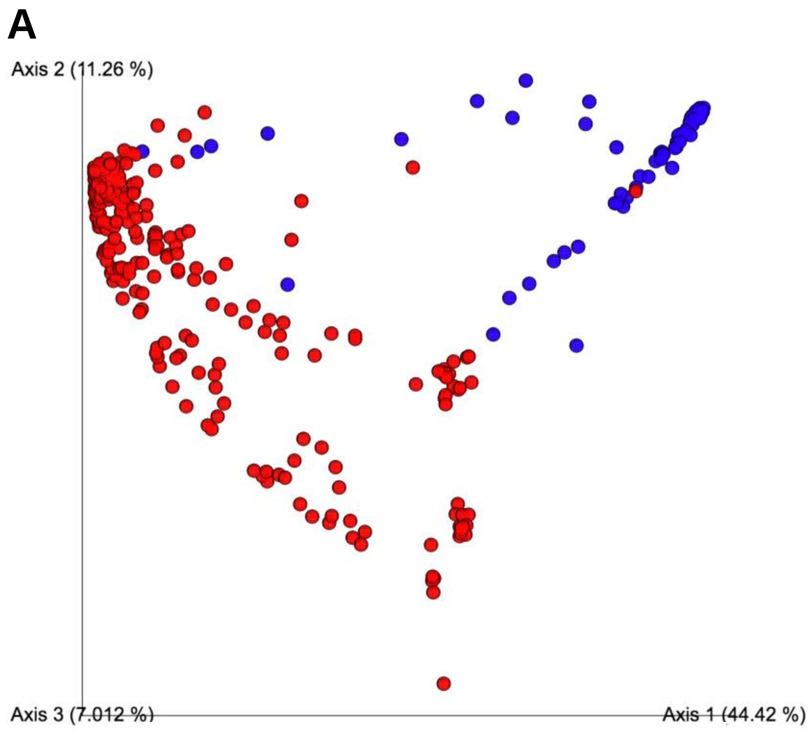

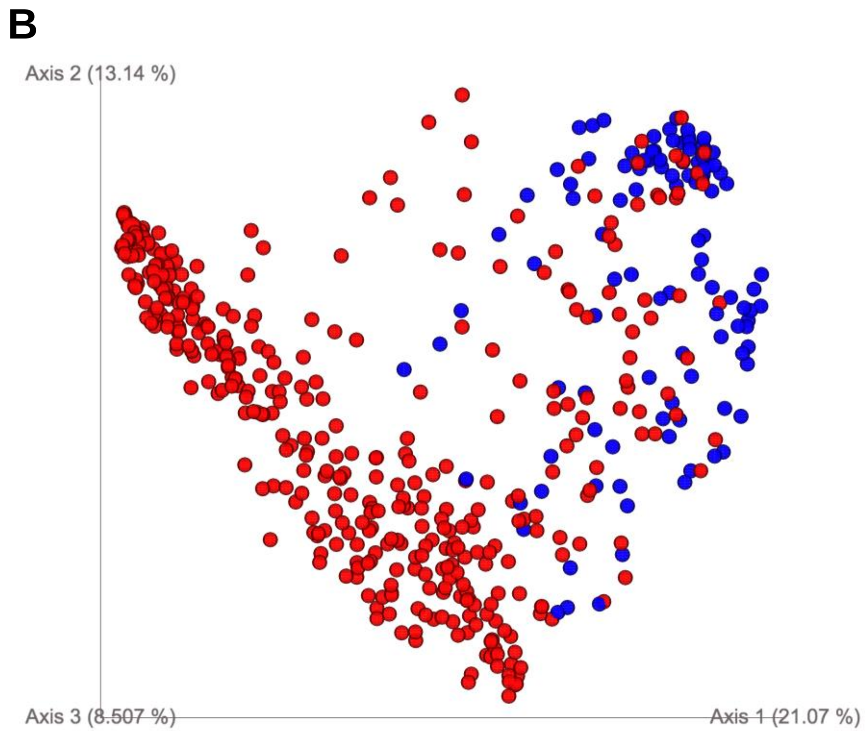

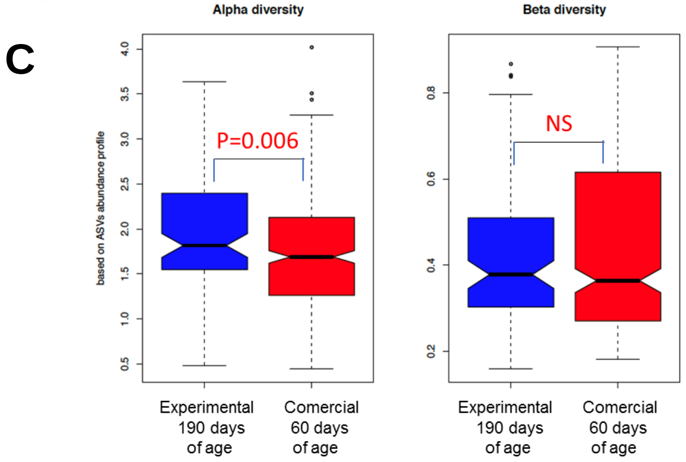

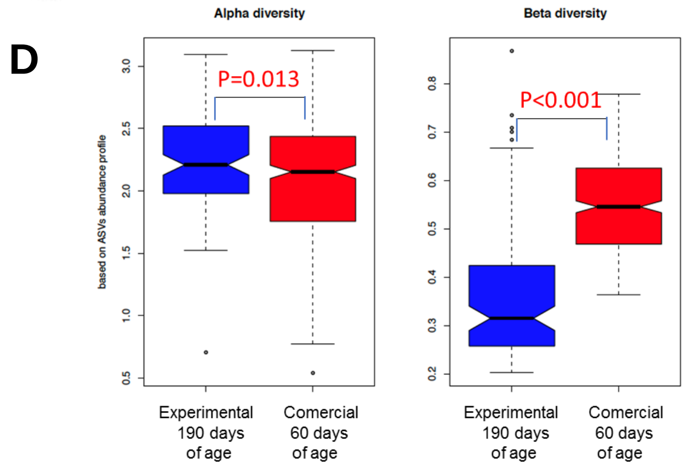

Supplement: Supplementary file 2 — Additional file 2 Figure S1. Sample distribution of fungi (A) and protist (B) communities. Diversity indexes of fungi (C) and protist (D) communities. Blue color represents the finishing pigs (experimental farm at 190 days) and red the weaned piglets (commercial farm at 60 days). [file 42523_2020_38_MOESM2_ESM.pdf]
