# Supplementary figures and images for: Gut eukaryotic communities in pigs: diversity, composition and host genetics contribution
Source: Anim Microbiome. 2020 May 7;2:18. doi: 10.1186/s42523-020-00038-4 (PMC7807704; doi:10.1186/s42523-020-00038-4)

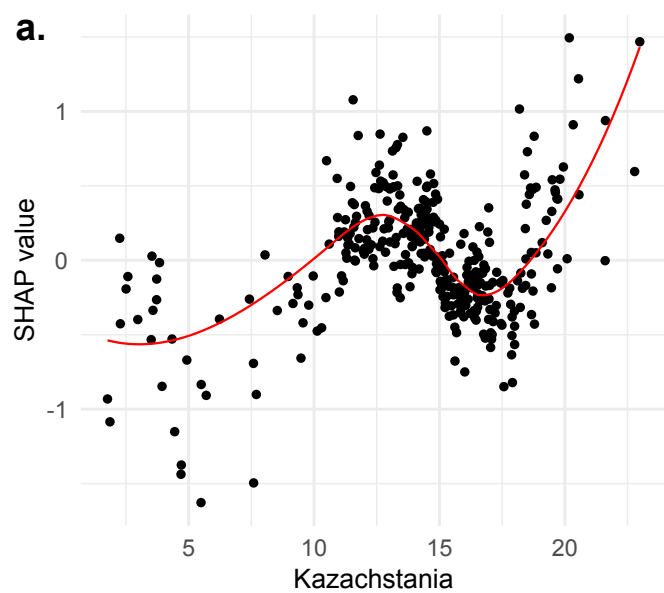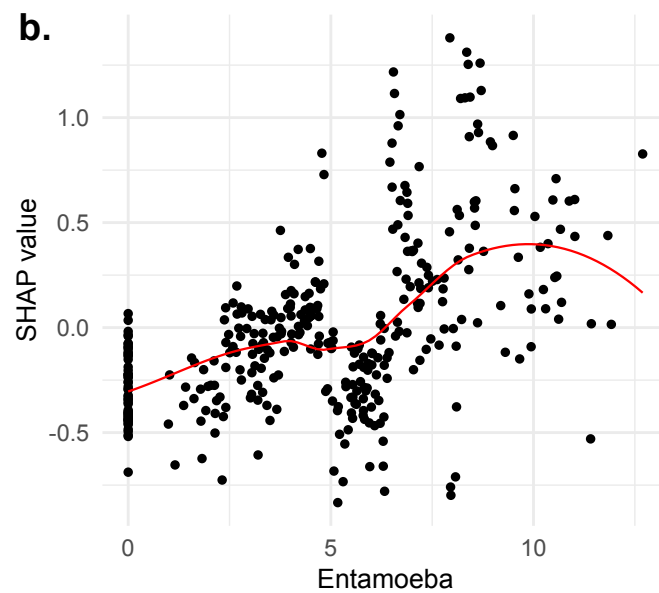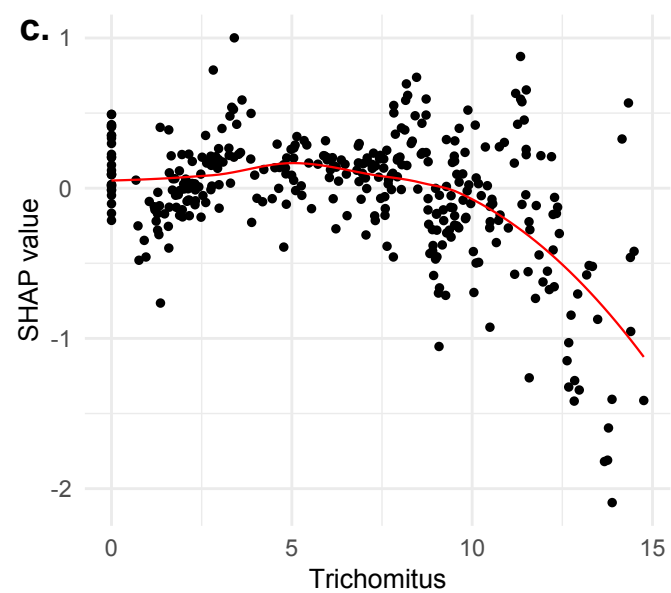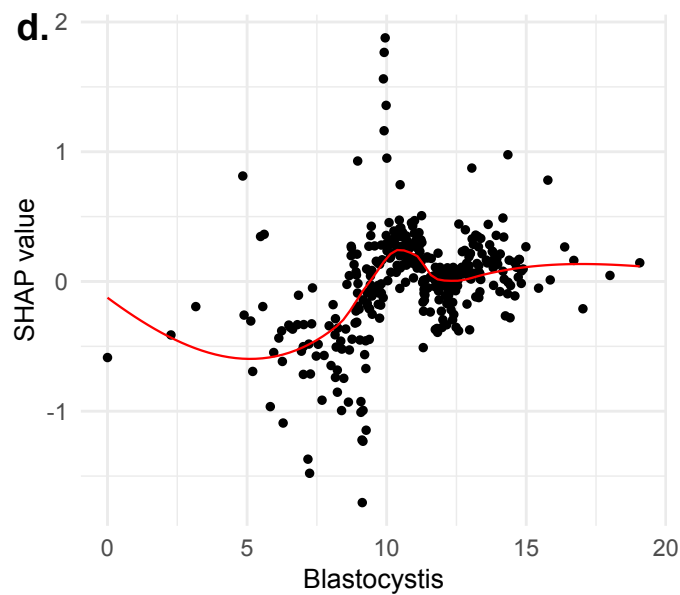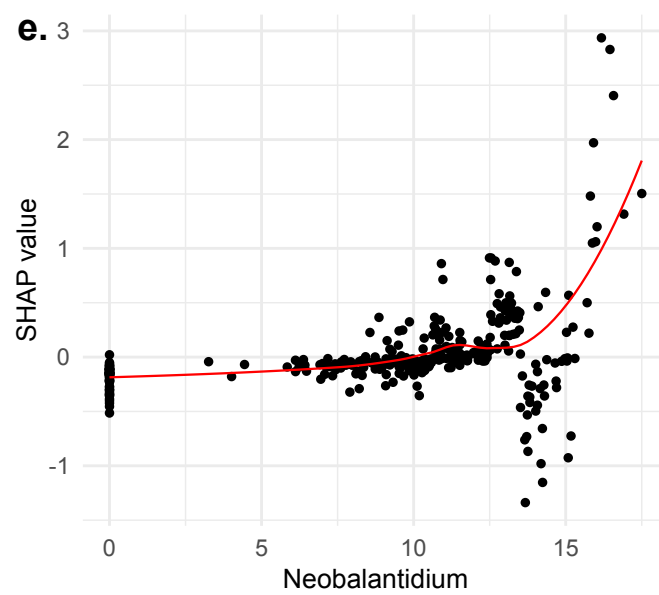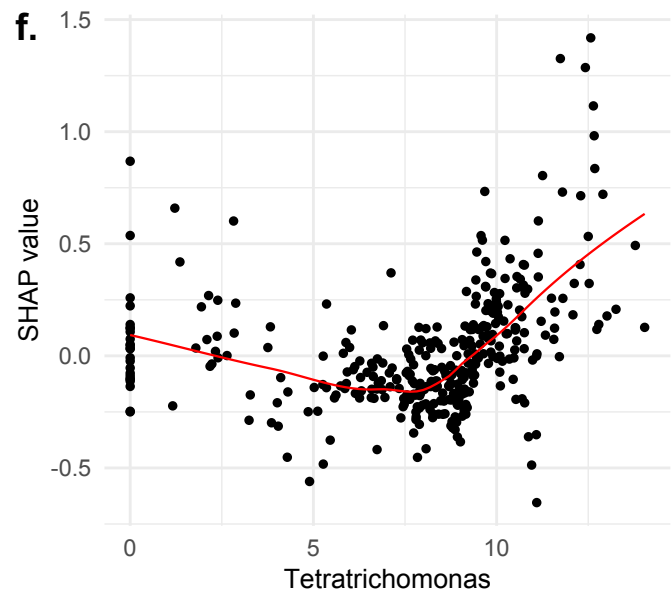

Supplement: Supplementary file 7 — Additional file 7 Figure S2. Patterns of association between the piglets body weight at 60-days old and the fungal and protist abundance according to the Shapley Additive Explanation (SHAP) values. The x-axis represent the genera abundance after Cumulative Sum Scaling (CSS) normalization and log transformation. Y-axis represents the SHAP values of the piglets’ body weight. [file 42523_2020_38_MOESM7_ESM.pdf]
